# Eating Competence and Dietary Intake of Sexual and Gender Minority College Students

**DOI:** 10.3390/nu13072388

**Published:** 2021-07-13

**Authors:** Sara Murphy, Jesse Stabile Morrell

**Affiliations:** Department of Agriculture, Nutrition and Food Systems, University of New Hampshire, Durham, NH 03824, USA; smurphy2195@gmail.com

**Keywords:** sexual and gender minority, eating competence, LGBT health

## Abstract

Sexual and gender minority college students are underrepresented in nutrition research and may face unique challenges related to eating which impact their overall diet quality. We assessed the differences in eating competence and dietary intake between sexual and gender minority (SGM) and cisgender heterosexual (CH) college students. Participants (*n* = 2645) reported sexual orientation, gender identity and completed the Eating Competence Satter Inventory (ecSI 2.0™ through an online questionnaire. Three-day food records examined dietary intake. Intake was compared to recommendations for nutrients of public health concern. Chi-square and ANCOVA examined differences between eating competence and dietary intake. There were no differences in total ecSI 2.0™ scores. Subscale scores for Eating Attitudes and Contextual Skills were significantly higher in CH vs. SGM students (13.4 ± 0.1 vs. 12.4 ± 0.4 *p* = 0.01 and 10.7 ± 0.1 vs. 9.9 ± 0.3, *p* = 0.01, respectively). Most students (40.8%) met one nutrient recommendation. The proportion of students meeting nutrient recommendations were similar for SGM and CH. SGM populations may struggle with attitudes and eating behaviors. Dietary intake of SGM and CH students were similarly inadequate when compared to recommendations.

## 1. Introduction

Lesbian, gay, bisexual, transgender, and queer identifying college students are an underrepresented population in nutrition research. These and other sexual and gender minority (SGM) populations may be at greater risks for unfavorable nutrition outcomes due to stigma, minority stress, and lack of access to healthcare [1,2]. More research is needed to better understand the trends identified in SGM youth and college students. The limited available research in SGM young adults consistently shows that some subgroups experience higher rates of unhealthy weight control behaviors, higher rates of eating disorders, and greater body dissatisfaction [3,4,5]. Laska et al. found that in a sample of 33,907 college students across Minnesota, using self-reported data to calculate BMI, sexual minority men were more likely than heterosexual men to be categorized as underweight (BMI < 18.5 kg/m^2^) [6]. They also found that sexual minority men were more likely to engage in disordered eating behaviors and had lower body satisfaction [6]. Utilizing data from the same College Student Health Survey, Simone et al. found that SGM students reported higher odds of having eating pathology-specific academic impairment [7]. Using data from the Massachusetts Youth Risk Behavior Survey, Watson et al. reported that sexual minority youth were more likely to use diet pills, fasting, and purging to lose weight, compared to heterosexual men and women [8]. In a national sample of college students, transgender students were four times more likely to be diagnosed with an eating disorder compared to their cisgender heterosexual (CH) women classmates, often the most stereotypical face of eating disorders [3]. Despite the rising number of young people identifying as SGM students, these findings suggest that SGM young adults are at risk of developing unhealthy eating behaviors and dieting practices and a better understanding is needed to help guide health educators and providers to affirming and evidence-based care.

One approach to quantify relationships with food and eating is by measuring someone’s eating competence. Eating competence describes an individual’s attitude and behaviors regarding dietary intake and can be quantified using the Satter Eating Competence Inventory (ecSI 2.0™) [9]. Someone who is eating competent is characterized as being “positive, comfortable, and flexible with eating and is matter-of-fact and reliable about getting enough to eat of enjoyable and nourishing food” [10]. The ecSI 2.0™ used to measure eating competence was first developed in 2007 by the Ellyn Satter Institute and has since been updated to increase validity and reliability [11]. Lower eating competence in college students was shown to be associated with poor sleep quality, higher rates of eating disorders, greater stress, higher BMI, greater desire to lose weight, and being female [12,13,14,15,16,17,18]. As these same factors have also been shown to be higher in SGM populations, characterizing the eating competence of this population may help elucidate the specific feelings towards food and eating behaviors and better understand the factors associated with these negative health outcomes.

Leaving home to attend university/college has long been recognized as a time of transition and independence for young adults. Students move away from the family home, experience new relationships, make independent decisions about lifestyle behaviors and time management, and make autonomous choices related to personal health, including dietary intake choices. While this new independence and self-autonomy is exciting, it often leads to less nutritious choices including a low intake of fruits and vegetables, low fiber, frequent intake of sugar sweetened beverages, and higher alcohol consumption [19,20,21]. Our primary objective of this study is to examine the differences in ecSI 2.0™ scores between CH and SGM college students. As there is limited research on the dietary behaviors of SGM populations, our secondary objective is to examine what percentage of CH and SGM students are meeting the dietary recommendations laid out in the 2015–2020 Dietary Guidelines for Americans [22]. Given that the percentage of college students that report their sexual orientation as something other than heterosexual has nearly doubled in the last five years, from 11.5% in 2015 to 22.1% in 2020, and the number of students that identify outside of the gender binary (male/female) has risen from 0.5% in 2015 to 3.8% in 2020, this research has the potential to aid campus administrators and health educators better meet the needs of their student population [23,24].

## 2. Materials and Methods

Data were collected between 2015 and 2020 from the College Health and Nutrition Assessment Survey (CHANAS), an ongoing, cross-sectional study at a public university in the northeastern United States. At the start of each semester, participants were recruited from a general education introductory nutrition course. This course is delivered via lecture and laboratory modalities and students represent all academic majors in the university. Participants were included if they were 18–24 years old, not pregnant, and did not have a medical condition that would inhibit them from safely participating in the study. Informed consent was collected from all participants prior to any data collection. The project was approved by the university’s institutional review board (IRB# 5524) prior to initiation and reviewed annually thereafter.

Demographic data were collected from an online survey (Qualtrics; Provo, Utah) sent out via email to students during the first quarter of the semester. Students had two weeks to complete the survey and were emailed reminders to encourage participation. Survey items queried participant demographics and covariates including self-reported gender, age, sexual orientation, participation in federal tuition aid programs (e.g., Pell Grant), and frequency of dining hall usage. The survey item on sexual orientation was phrased “Do you consider yourself to be:” with options of “heterosexual or straight”, “gay or lesbian”, “bisexual”, “other”, and “I choose not to answer”. Participants were considered a sexual minority if they identified as gay or lesbian, bisexual, or “other” and were considered a gender minority if they identified as transgender, nonbinary, or “other”. Participants that chose not to answer sexual orientation or gender identity items were excluded from our analyses. Prior to the midsemester timepoint, participants attended a health risk screening where anthropometric and biochemical data were collected after an overnight fast. Height and weight measurements were measured in duplicate by trained personnel using calibrated, digital scales and stadiometers. Mean values were used to calculate BMI (kg/m^2^).

Eating competence was measured as part of the online survey using the Satter Eating Competence Model (ecSI 2.0™), a validated 16-item questionnaire that is divided into four subscales: Eating Attitudes (EA), Internal Regulation (IR), Food Acceptance (FA), and Contextual Skills (CS) [9]. Responses for each item were collected via Likert scale ranging from always to never. Total eating competence scores ranged from 0–48. Possible subscale scores are 0–18 (EA), 0–6 (IR), 0–9 (FA), and 0–15 (CS) [11]. An individual is categorized as “eating competent” when they receive a total ecSI 2.0™ score of 32 or higher (i.e., >32).

Approximately halfway into the semester, participants were asked to complete a food record for three non-consecutive days: two weekdays and one weekend. Participants were educated on portion estimation via in-class lesson, provided reminders to increase the accuracy of their dietary intake (e.g., record preparation methods, include frequently overlooked items), and prompted to review errors for completion. Participants then entered their food records into an online nutrient analysis program (Diet Analysis+, v10, Diet and Wellness+, Cengage Learning) and average daily nutrient, energy, and fiber intakes were obtained. Participants were considered to meet the 2015 Dietary Guidelines for Americans (DGA) recommendations if their daily averages met the following: <10% of total calories from saturated fat, <2300 mg of sodium, fiber intake ≥25.2 g/day for women and ≥30.8 g/day for men, calcium intake ≥1300 mg for 18-year-old participants and ≥1000 mg for all other participants, potassium ≥4700 mg/day, and vitamin D intake ≥15 mcg/day [22]. Selected nutrients were chosen as they are considered nutrients of public health concern in the 2015 Dietary Guidelines for Americans [22].

Prior to analyses, all data were screened for errors, abnormalities, and outliers. Data were corrected if veracity could be confirmed or removed when recorded data were implausible. Data are presented as frequencies or means ± standard error. Independent sample T-test was used to compare age between groups. Chi-square test of homogeneity was used to examine differences in meeting DGA recommendations and eating competence status between CH and SGM students. Mean differences between CH and SGM students in total ecSI 2.0™ scores, subscale scores, nutrient intake, and BMI were examined using ANCOVA with covariates of gender, age, dining hall usage, and Pell Grant recipient status; total kilocalories and BMI were also included as covariates for nutrient intake comparison. Analyses were completed with and without the Bonferroni correction, however, as no substantive differences were observed, data are presented without adjustment. Levene’s test was used to assess the homogeneity of variance due to large differences in sample size between the CH and SGM groups. Levene’s test showed differences in homogeneity of variance between groups for BMI, Eating Attitudes subscale, and Internal Regulation subscale. Analyses were preformed using IBM SPSS Statistics 26^®^ (Chicago, IL, USA) and significance was established as *p* < 0.05.

## 3. Results

Eighty-nine percent (*n* = 2900) of the students enrolled in the course (*n* = 3251) provided written, informed consent at the start of the term. Of these participants, 4.7% (136) were excluded due to the age parameters of the study, 4.1% (*n* = 119) were excluded due to missing data on gender identity or sexual orientation. If students had answered some, but not all ecSI 2.0™ survey items, subscales scores were still calculated when possible, however, total scores were not. Data were collected from a total of 2645 subjects (participant characteristics in Table 1).

Participants mostly identified as heterosexual (96.2%), white (93.4%), and female (64.6%). Approximately one-quarter (21.4%) of students reported receiving a Pell Grant, however, 18.2% were unaware if they received one or not. Only 3.8% (*n* = 100) of the sample identified as a sexual minority and 0.2% (*n* = 6) identified as a gender minority. Four of the six people that identified as transgender also identified as sexual minorities. Most students (77.0%) were 18 or 19 years old and most (70%) had a BMI between 18.5–24.99 kg/m^2^. The mean ecSI 2.0™ score of the sample was 33.42 ± 8.9 and more than half (60.2%) met the definition of eating competent. CH students tended to have a higher mean ecSI 2.0™ scores vs. students who identify as a SGM (Table 2) and tended to be considered eating competent compared to SGM students (60.6% vs. 52%, *p* = 0.09). Mean subscale scores for EA and CS were significantly higher in CH vs. SGM students (13.4 ± 0.1 vs. 12.4 ± 0.4, *p* = 0.01 and 10.7 ± 0.1 vs. 9.9 ± 0.3, *p* = 0.01, respectively); there were no differences in the subscale scores for FA or IR between CH students vs. SGM students.

**Figure 1:**
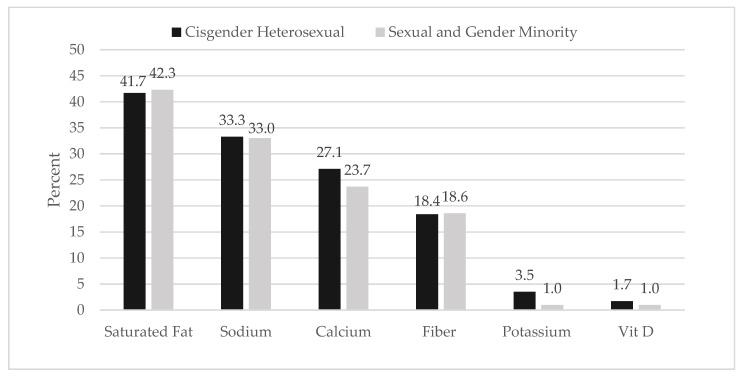
Percent of students that met nutrient recommendations by sexual orientation and gender identity.

Overall, very few students met the six DGA recommendations evaluated; only 2.2% (*n* = 56) met >4 DGA recommendations evaluated. The majority (40.8%) of students were only meeting one of the six DGA recommendation; about one-quarter (23.5%) of students failed to meet any of DGA recommendations evaluated. Students were most likely to meet recommendations for saturated fat and sodium (41.7% and 33.3%, respectively), as compared to calcium and fiber (27.0% and 18.5%, respectively); very few students met the recommendations for potassium or vitamin D (3.5% and 1.7%, respectively). There was no significance difference in the percentage of SGM vs. CH students that met zero recommendations (25.8% and 23.4%, *p* = 0.7). Furthermore, a similar percentage of CH and SGM students were meeting > 3 recommendations (10.9% vs. 9.3%, *p* = 0.7). Differences between SGM and CH in meeting recommendations for specific nutrients and fiber were not significant (Figure 1).

## 4. Discussion

To our knowledge, this is the first time that eating competence has been looked at in the context of sexual orientation and gender identity. Our findings suggest modest differences in eating competence, specifically the Eating Attitudes and Contextual Skills subscale, between SGM students and CH college students. While total ecSI 2.0™ scores did not differ in our sample, this study adds to the existing research which suggest that SGM populations may face unique challenges with attitudes and eating behaviors. Furthermore, this study highlights the uniform inadequacy of students’ diets.

Other eating competence research that focused on college students has put mean ecSI 2.0™ scores below 32, the score at which people are considered eating competent, indicating that college students as a whole may struggle with attitudes and behaviors around eating, meal planning and food flexibility [12,13,14,15,17,18]. Our unadjusted sample mean ecSI 2.0™ score was 33.42 ± 8.9 with 60.2% of our total sample considered eating competent. Higher mean scores compared to other research could be because of the high proportion of our first-year students in our sample (50.1%). As first year students from this institution are generally required to live on campus and pre-purchase meal plans which give them unlimited access to buffet style dining halls, dining hall access and utilization may explain the higher eating competence score in this sample [25]. The use of dining halls increases food security, decreases the number of decisions one makes about what to eat, and decreases the amount of planning and time that goes into making meals [26].

Our findings illustrate distinctions in the ecSI 2.0™ subscales between SGM and CH students: Eating Attitudes (EA) and Contextual Skills (CS). Each subscale of the ecSI 2.0™ aims to quantify different aspects of eating. The EA subscale aims to capture someone’s feelings towards food and eating, both internal and external food experiences, and a harmony between what someone wants to eat, what foods they choose to eat, and how much they eat [9]. This subscale was significantly lower for SGM students. This could indicate that SGM students have greater negative emotions around eating and food experiences. While we did not collect data on history of eating disorders, other research has shown that college students that previously had or currently have an eating disorder also scored significantly lower on this subscale [12]. The CS subscale was also significantly lower for SGM students. This subscale focuses on the skills needed to plan for and prepare regularly scheduled, nourishing meals. Managing meals and eating depends on discipline and prioritizing one’s physical needs, but also engagement with thoughts around food. SGM students scored significantly lower on this subscale, indicating a possible avoidance of thoughts centered around mealtimes and meal planning. As negative attitudes centered around meals and a lack of planning/scheduling regular, nourishing meals can lead to unhealthy eating behaviors, further exploratory research in the SGM population is warranted. For example, interviews or focus groups may help elucidate potential barriers to carrying out and planning nourishing means and may also pinpoint specific attitudes or emotions around eating.

The lack of differences seen between the SGM and CH students for the Food Acceptance subscale could be attributed to the convenience sample of students from a nutrition class. Although the general education course included students from all majors across the university, enrollment may have been liked to pre-existing interest in food and therefore the participants may have been more flexible with the types of foods they eat than other individuals. We also saw no difference in SGM and CH students on the Internal Regulation subscale. One reason we may have not seen a difference is that college students may rely more heavily on their schedules than internal cues to dictate how much or little they are eating during a meal or snack. For example, a student that has classes back-to-back from mid-morning until the early afternoon may be eating a larger breakfast than is physically comfortable to hold them over until their next break between classes.

The secondary objective of this study was to assess and compare the diets of SGM students to their CH counterparts. We found that, overall, the college students from this sample were not meeting the DGA recommendations regardless of sexual orientation or gender identity. There is inconsistent evidence on the diet quality of the SGM population. Some researchers have found that despite higher rates of eating disorders and obesity, sexual and gender minority adolescents (10–23 years old) report higher intake of fruits and vegetables and have higher diet quality scores compared to CH adolescents [27,28]. Conversely, in a study utilizing data from the National College Health Assessment Survey with a total of 18,440 participants found there was no difference in fruit and vegetable consumption between sexual minority women and heterosexual women [29]. However, this national survey only queries whether students are eating five servings of fruits and vegetables per day and did not complete any food records, FFQs, or 24 h recalls. Laska et al. also looked at the percentage of students eating greater than or less than five servings of fruits and vegetables per day [6]. They found that there were no differences by sexual orientation among males in the percentage of students eating less than five servings/day [6]. For females, a greater percentage of those identifying as lesbians were eating less than five servings/day compared to bisexual women, however, there was no statistical difference compared to heterosexual women [6]. Our study adds to this existing literature by assessing dietary intake in a new way by using a 3-day food record. What is consistent between the findings from other studies and ours is that overall, college students are not meeting dietary recommendations, and therefore, could benefit from effective nutrition education and/or intervention. The evidence reported to date is unclear on whether SGM students are at greater risk of nutrition inadequacy. Further research on sexual minority populations and dietary intake measured by 3-day food records, a series of 24 h recalls, or validated FFQs is needed to ensure that interventions with this population are specific, relevant, and effective to improve dietary intake.

The most recent report from the National College Health Assessment shows that 3.8% of college students reported that they identify outside the gender binary [24]. This study only had six students that identified as gender minorities, which made up 0.2% of our sample. Four of the six students that identified as a gender minority also identified as a sexual minority. In fact, our sample of sexual minority students was much lower than what the National College Health Assessment reported, 3.2% compared to 22.1% [24]. Although we cannot be sure why these numbers are low, it could be because the majority of the students in the sample were first-year students enrolled in their first semester. Younger students or students adjusting to the new campus environment may not yet feel comfortable identifying as an SGM student. There could also be more financial barriers for SGM populations which affect their enrollment at this four-year, public university. Future research could focus on recruiting SGM populations who come from diverse backgrounds, socioeconomic situations, and education levels. Other unidentified factors keeping SGM students from enrolling in this introductory nutrition course could also be at play (i.e., interest in other courses, the avoidance of nutrition specifically).

The major strength of this study is that it begins to chip away at the large gap of underrepresentation of minority populations in nutrition research and provides further context to the existing research on SGM populations. This study is the first to the best of our knowledge that looks at eating competence in the SGM population and our hope is that it will provide a basis for targeted interventions or nutrition education.

These findings provide a rationale for future research to better understand the challenges and support needed to initiate healthy eating among SGM college students. Nonetheless, our study’s limitations should be considered. First, the cross-sectional data were obtained from a convenience sample, and therefore, causality cannot be established. In addition, while the study gathered detailed dietary data from 3-day food records, participants entered their own food intake into the analysis software. Though participants were trained on portion sizes and estimation as well as how to enter substitutions, errors, and/or eliminations from the food records on certain items not in the database could have occurred and increased the likelihood of recording mistakes. Furthermore, it is important to address the limitation of grouping all sexual and gender minority students together as one SGM group. While almost all our gender minority students also identified as sexual minorities, it must be noted that these groups face unique challenges and experiences which do not always intersect. Being that our sample of transgender/nonbinary students was small, we decided to collapse the group to keep participants from being identifiable. Finally, our sample was predominately white and female, and thus findings may not be generalizable to other populations.

## 5. Conclusions

As more young people on college campuses are identifying as SGM students, it is important to assess the needs of this community and target appropriate education and interventions to address health disparities. Our work showed differences in eating competence at a subscale level between SGM and CH college students. These findings will assist health professionals develop targeted and affirming evidence-based resources and standards of care. We hope that this research will prompt further research from diverse campuses to develop appropriately targeted nutrition education and support for a diverse student body.

## Figures and Tables

**Table 1:** Characteristics by sexual orientation and gender identity.

	Cisgender Heterosexual (*n* = 2538)	Sexual and Gender Minority (*n* = 102)	*p* Value
**Gender**, *n* (%)			
Male	909 (35.8)	20 (19.6)	<0.001
Female	1629 (64.2)	76 (74.5)	
Transgender/nonbinary	0	6 (5.8)	
**Age**, years	19.0 ± 0.02	19.4 ± 0.1	
**BMI**, kg/m^2^	23.46 ± 0.08	24.93 ± 0.38	0.004
**Pell Grant Recipient**, **%**			<0.001
Yes	21.1	28.4	0.14
No	59.2	58.8	
I do not know	18.4	12.7	
I choose not to answer	1.3	0.0	
**Dietary Intake**			
Kcals	1950.3 ± 12.5	1972.9 ± 63.2	
Fruits (cup eq)	1.12 ± 0.02	1.08 ± 0.10	0.72
Vegetables (cup eq)	1.79 ± 0.03	1.76 ± 0.13	0.63
Grains (oz)	6.33± 0.05	7.02 ± 0.22	0.84
Dairy (cup eq)	1.75 ± 0.02	1.65 ± 0.12	0.003
Protein (oz)	7.93 ± 0.09	6.93 ± 0.47	0.40
Discretionary Kcals	562.6 ± 4.2	590.0 ± 21.3	0.04
Saturated fat (g)	23.29 ± 0.14	22.86 ± 0.69	0.21
Sodium (mg)	2964.09 ± 17.20	2963.22 ± 86.92	0.54
Calcium (mg)	917.79 ± 6.95	903.37 ± 35.14	0.99
Fiber (g)	20.29 ± 0.17	21.00 ± 0.86	0.69
Potassium (mg)	2343.86 ± 16.45	2319.44 ± 83.16	0.42
Vit D (mcg)	3.98 ± 0.06	3.91 ± 0.31	0.77
Cholesterol (mg)	338.56 ± 4.07	283.50 ± 20.57	0.83

Continuous variables are presented as mean ± standard error.

**Table 2:** Eating competence by sexual orientation and gender identity.

	Possible Score	Cisgender Heterosexual (*n* = 2352–2405)	Sexual and Gender Minority (*n* = 95–97)	*p* Value
**Total Score**	0–48	33.6 ± 0.2	32.0 ± 0.9	0.06
Eating attitudes	0–18	13.4 ± 0.1	12.4 ± 0.4	0.01
Food acceptance	0–9	5.3 ± 0.1	5.5 ± 0.3	0.50
Internal Regulation	0–6	4.2 ± 0.03	4.1 ± 0.1	0.38
Contextual skills	0–15	10.7 ± 0.1	9.9 ± 0.3	0.01

Continuous variables are presented as mean ± standard error.

## Data Availability

Data presented in this study are available on request from the corresponding author.
